# Autistic adults’ experiences of cognitive-behavioural group therapy for social anxiety: Relational experiences of participation

**DOI:** 10.1177/13623613251377930

**Published:** 2025-10-18

**Authors:** Bruna B Roisenberg, Kelsie A Boulton, Emma E Thomas, Nina Perry, Dorothy Yu, Adam J Guastella

**Affiliations:** 1 The University of Sydney, Australia; 2 Child Neurodevelopment and Mental Health Team, Brain and Mind Centre, University of Sydney, Australia

**Keywords:** mental health, peer connection, psychological intervention, thematic analysis, treatment

## Abstract

Autistic adults often report high levels of social anxiety and significant life impacts from this anxiety on well-being and functioning. Although cognitive-behavioural therapy in group settings has been adapted for autistic populations, there is a paucity of research exploring how autistic individuals experience CBT interventions. This qualitative study investigated autistic adults’ experiences of a modified cognitive-behavioural therapy group programme for social anxiety. Twenty-seven autistic participants were recruited through clinical services and community outreach. Following the intervention, participants completed semi-structured individual interviews about their experience of the programme. Participants reported both therapeutic benefits and challenges following their participation. Themes included reflections on group dynamics, mental health impacts, social functioning and daily life applications of strategies. Peer connection emerged as a core facilitator of engagement, while structured strategies were credited with bolstering social confidence and self-awareness. However, challenges, such as sensory sensitivities, the role of previous therapeutic experiences and difficulties applying strategies beyond the therapy context, were also highlighted.

**Lay Abstract:**

Autistic adults often report high levels of social anxiety. To support autistic people with social anxiety, we developed a group therapy programme, the Engage Program, an intervention designed specifically for autistic adults. Our study aimed to better understand what it is like for autistic adults to take part in group therapy and what aspects of the programme were most helpful, or not. The programme aimed to provide a safe space where participants could explore social interactions in a way that felt comfortable and authentic. Rather than encouraging people to hide or ‘camouflage’ their autistic traits, the programme focused on building confidence, self-understanding and meaningful connections with peers. Sessions included guided discussions, structured activities and shared experiences. We interviewed participants who had completed the group programme to hear directly from them about their experiences. Many said they felt less alone and more socially confident after taking part in the group. They appreciated connecting with others who understood their experiences, and they said this helped them feel more accepted and supported. They also reported that participating in the group helped them understand their social strengths and challenges more clearly. However, not everything worked for everyone. Some participants found aspects of the therapy challenging, especially if they had sensory sensitivities or difficult past experiences with therapy. In the future, we want to explore how to make group therapy more flexible and accessible, especially for those who may struggle in traditional settings. This research shows that group therapy can be a powerful way to support autistic adults, especially when it is designed with their needs and preferences in mind.

## Background

Autistic individuals face disproportionately high rates of mental health difficulties, including elevated levels of social anxiety compared to the general population (Boulton & Guastella, 2021; Hollocks et al., 2018; Lai et al., 2019; Van Steensel et al., 2011). The core components of social anxiety, such as fear of negative evaluation and avoidance of social situations, can further interact with social communication differences that autistic individuals often experience to further impact quality of life and functioning (Hull et al., 2021; Lei, Mason, Russell, Hollocks, & Leigh et al., 2024; Woolard et al., 2021).

Although research on mental health in autistic children has advanced, the unique needs of autistic adults with social anxiety remain underexplored (Spain et al., 2016; Wilson & Gullon-Scott, 2024). There is a growing need to identify effective and accessible support strategies tailored to this population. Cognitive-behavioural therapy (CBT) is an evidence-based intervention that has demonstrated effectiveness in reducing social anxiety among autistic adults (Bemmer et al., 2021; Cervin et al., 2023; Roisenberg et al., 2025; Weston et al., 2016). CBT is the gold standard treatment for social anxiety in the general population (Heimberg, 2002) and has been increasingly adapted to meet the needs of autistic individuals. Although the evidence base is still developing, modified CBT approaches that consider the sensory and communication needs of autistic adults have shown promise in reducing anxiety and enhancing social confidence (Bemmer et al., 2021; Spain et al., 2017). However, there remains limited qualitative research exploring how autistic individuals experience these adapted, group-based interventions (Lei, Cooper, & Hollocks, 2024).

There is an ongoing debate surrounding the use of traditional behavioural therapies, including CBT, for autistic individuals. Critics argue that these approaches, often rooted in neurotypical social norms, risk prioritizing conformity and camouflaging behaviours over authentic self-expression (Bottema-Beutel et al., 2018). There is also concern about exposure tasks and cognitive restructuring not taking into consideration real negative experiences, such as bullying and exclusion, that are often reported by autistic people (Wilson, 2024). One avenue to mitigate some of these processes is through group-based therapies. Some of the potential benefits of group-based programmes may lie in the opportunities for peer support (Bemmer et al., 2021; White et al., 2018). Interacting with other autistic individuals in group situations might validate shared experiences, reinforce feelings of safety, as well as reduce social pressures and improve inclusion in social situations (Crompton et al., 2020). Emphasizing neurodivergent community and relational approaches in group therapy can create supportive environments that prioritize authentic interactions, offering meaningful therapeutic outcomes (Bottema-Beutel et al., 2018; Mitchell et al., 2021). However, despite growing evidence supporting these adaptations, the personal experiences and perspectives of autistic individuals participating in modified CBT programmes, especially targeting social anxiety, remain underexplored.

This study is one of two papers examining the experiences of autistic adults who took part in a modified CBT group intervention for social anxiety. Although both papers explore participant perspectives, they vary in their emphasis and scope. This article focuses on group dynamics and the role of peer interactions in shaping participants’ therapeutic journey, including perceived benefits and challenges of the group therapy process. We use a qualitative approach to identify themes related to each of these factors. Given the scarce research in this area, we did not make predictions about specific themes that would emerge. In addition, the companion paper focuses specifically on participants’ experiences with specific CBT components used in the programme, such as cognitive restructuring, behavioural experiments and exposure tasks and how autistic individuals perceived the benefits and limitations of these methods. A separate quantitative study investigates how social anxiety symptoms and camouflage reports change following this intervention (Roisenberg et al., 2025). We found significant reductions in social anxiety symptoms, with a trend towards reduced camouflaging behaviours. Interestingly, this reduction in camouflaging behaviour was associated with more social anxiety symptom reduction following the CBT intervention.

## Method

### Participants

Twenty-seven participants were recruited through clinical referrals, community outreach and local headspace centres from May 2023 to July 2024. Participants were required to have a formal diagnosis of autism spectrum disorder. For individuals diagnosed within the previous 12 months, diagnostic documentation was accepted as provided. For those diagnosed more than 12 months before the study, diagnostic verification was conducted using the Autism Diagnostic Observation Schedule-2 (ADOS-2) as part of the research protocol. This ensured consistency in diagnostic criteria across the sample, regardless of when the original diagnosis was received. All participants were seeking support for social anxiety symptoms and met diagnostic criteria for social anxiety disorder based on the Anxiety Disorders Interview Schedule (ADIS). Additional eligibility criteria included an age of 16 or older, a Full-Scale IQ of at least 70 (assessed using the Wechsler Test of Adult Reading; WTAR) and no evidence of active psychosis, acute mental health issues, high suicide risk, severe depression, substance abuse or significant sensory impairments (e.g. vision or hearing) that could affect engagement with the intervention’s audio/visual components. Participants were not excluded based on prior or current psychological or pharmacological treatments.

The final sample had a mean age of 25.2 years (SD = 5.09), ranging from 18 to 38 years old, and a mean IQ score of 113 (SD = 5.84). The group included diverse gender identities, with 29.6% identifying as male, 44.4% as female, 14.8% as other and 11.1% preferring not to disclose.

At baseline, participants completed the Liebowitz Social Anxiety Scale (LSAS) (Kanai et al., 2011; Liebowitz, 2015), a validated measure of social anxiety in autistic adults. The sample showed high levels of social anxiety, with a mean LSAS total score of 82.50 (SD = 29.13). Subscale scores indicated substantial fear (M = 42.00, SD = 16.15) and avoidance (M = 40.50, SD = 13.99) in social situations. These scores fall within the moderate to severe range of social anxiety, consistent with clinical levels. In addition to the LSAS, participants completed a battery of measures assessing broader psychological, social and functional characteristics. On the Kessler Psychological Distress Scale (K10) (Kessler et al., 2003), participants reported high levels of distress (M = 29.04, SD = 7.85). On the Depression Anxiety Stress Scales (DASS-21; Park et al., 2020), mean raw scores were 33.05 for depression, 14.92 for anxiety and 23.92 for stress, indicating moderate to high levels of emotional difficulties. Quality of life, as assessed by the WHOQOL-BREF (WHOQOL Group, 1998), was reduced across all domains, with particularly low scores in the psychological domain (Domain 2: M = 35.26, SD = 13.76). WHODAS 2.0 (Üstün et al., 2010) scores reflected considerable functional impairment (total score: M = 81.39, SD = 22.93). Participants also reported elevated levels of camouflaging behaviours as measured by the Camouflaging of Autistic Traits Questionnaire (CAT-Q; Hull et al., 2019) (CAT-Q Total: M = 120.31, SD = 30.56) and high social communication difficulties as measured by the Social Responsiveness Scale-2 – Adult Self-Report (SRS-2; Constantino & Gruber, 2012) (SRS-2 SCI: M = 94.62, SD = 19.77; total score: M = 114.27, SD = 25.65). Overall, these scores reflect the complex clinical presentations and support needs of the participants at the start of the intervention.

### Materials

#### Semi-structured interview

The study authors designed a semi-structured interview to explore participants’ experiences with the group therapy intervention. Interview questions were developed by a professor of clinical psychology with over 20 years of experience in research and clinical practice, and a PhD candidate with a master’s degree in mental health and more than 7 years of experience as a clinical psychologist. These questions were crafted to gather detailed feedback on the overall group experience. Questions were developed to understand how group dynamics, peer interactions and shared learning shaped participants’ experiences (see Table 1). The interview structure provided flexibility, allowing participants to elaborate on both the benefits and challenges of the programme while highlighting the broader impact of group therapy on their social confidence and well-being (a copy of the interview is in the supplementary materials).

**Table 1.** Aspects of the group and corresponding example questions from the interview.

Aspects of the group experience	Example interview question
Overall group experience	What was your experience with the group therapy?
	What were the most valuable aspects of the group for you?
Areas for improvement	Were there any aspects of the group that you found less helpful or felt could be improved?
Psychoeducation	How useful did you find the education about autism and anxiety as part of the therapy?
Group dynamics	How important was being part of a group with other members?
	Did the group dynamic influence your experience in any way?

The whole interview can be found in the supplementary materials.

#### Programme development

This qualitative study examined a modified CBT programme tailored for autistic adults experiencing social anxiety. The intervention, known as the Engage Program (Bemmer et al., 2021; Roisenberg et al., 2025), was informed by established CBT protocols for social anxiety (Heimberg, 2002; Hofmann & Otto, 2008; Rapee et al., 2009) and adapted to meet the specific needs of autistic participants. Modifications included structured goal setting and the integration of social communication strategies, such as initiating and exiting conversations, setting boundaries and navigating assertive interactions. These components were not intended to teach normative social behaviours, but rather to provide scaffolding for exposure-based tasks and support participants in experimenting with approaches to social engagement that aligned with their individual goals and needs. This structure aimed to enhance participants’ confidence and facilitate meaningful engagement with the therapeutic process.

#### Modified CBT programme

The modified CBT programme was developed based on established guidelines for addressing anxiety in autistic adults (Ainsworth et al., 2020; Bemmer et al., 2021; Gaus, 2018; Spain et al., 2017). Delivered over 8 weeks, the programme consisted of weekly group sessions lasting 3 hours and included six to eight participants per group. Each weekly session focused on a specific topic, including psychoeducation about social anxiety and autism, goal setting, cognitive restructuring, behavioural experiments and exposure-based activities (see Table 2). The programme incorporated several adaptations designed to meet the needs of autistic adults. These included the use of clear, concrete language; visual supports (e.g. slides and worksheets); a predictable session structure; gradual pacing of exposure tasks; and opportunities for peer interaction in a supportive environment. Sensory needs were considered by ensuring low-stimulation settings, and participants were encouraged to use tools such as noise-cancelling headphones or to take breaks if needed. Individual communication and adaptation preferences were discussed during participants’ initial assessments, and adjustments were made throughout the programme in response to emerging needs. For example, participants could choose preferred communication modes (e.g. speaking, writing) during group activities, and facilitators regularly checked in to ensure comfort and accessibility. All sessions were delivered in person at a university-affiliated clinic.

**Table 2.** Outline of core CBT components across the 8-week programme.

Session	Core components
1	- Introduction to the programme
	- Group Guidelines
	- The anxiety response
	- Avoidance behaviours
	- Exposure guidelines
	- Homework allocation and Café time (skills practice/exposure)
2	- Recap and homework review
	- Sensory overwhelm and spoon theory
	- Trading information/starting conversations
	- Homework allocation and café time (skills practice/exposure)
3	- Recap and homework review
	- Social strategies for conversations
	- Homework allocation and café time (skills practice/exposure)
4	- Recap and homework review
	- Negative thoughts
	- Behavioural experiments/café visit
	- Homework allocation and café time (skills practice/exposure)
5	- Recap and homework review
	- Behavioural experiment
	- Anxiety management strategy
	- Exiting conversations
	- Homework allocation and café time (skills practice/exposure)
6	- Recap and homework review
	- Speech task
	- Homework allocation and café time (skills practice/exposure)
7	- Recap and homework review
	- Assertive communication/being tactful
	- Healthy relationship boundaries
	- Anxiety management: grounding
	- Homework allocation and café time (skills practice/exposure)
8	- Recap and homework review
	- Conflicts and handling disagreements
	- Relapse prevention
	- How to keep practising

Each session followed a consistent structure, beginning with a review of homework tasks from the previous week and a discussion of the current session’s focus. Homework assignments included activities such as initiating social interactions, tracking thoughts and emotions, and engaging in social situations to practice learned strategies. Sessions emphasized practical strategy development, including managing social anxiety, practising conversational strategies and exploring interpersonal engagement through hands-on exercises such as role-playing and behavioural experiments. The aim was not to teach participants to conform to normative standards of social behaviour, but to support them in experimenting with approaches to social interaction that aligned with their individual goals, preferences and needs.

At the end of each session, participants were introduced to their homework for the upcoming week. The final 30 min of each session were dedicated to ‘coffee time’, a practice period where participants could socialize in a supportive, real-life setting. During this time, facilitators provided individual guidance, helping participants plan homework and address personal challenges. More details on the group’s structure can be found in Table 2.

The programme was facilitated by two clinicians with expertise in autism and anxiety, who conducted a 60-minute debrief after each session to assess participant progress and refine goals for the following week.

#### Data availability statement

Materials used in this study (e.g. interview schedule, thematic codebook and analysis notes) are available via an anonymised OSF view-only link: https://osf.io/8u9ge/

All files have been de-identified.

### Procedures

#### Interviews

The qualitative data for this study were collected through individual, in-person interviews, allowing for an in-depth exploration of participants’ experiences with the group therapy programme. Interviews were conducted during the final week of the 8-week group intervention. Of the 46 participants who completed the programme and were invited to provide feedback, 27 consented to participate in the post-intervention interviews. All interviews were conducted in person in a private setting at the university. This timing allowed participants to reflect on their full experience of the programme while still being actively engaged in its final stages. The interviews were led by a PhD candidate specializing in clinical psychology and autism, with extensive experience in mental health and qualitative research. Audio recordings were made with participants’ consent, and the interviewer also took detailed notes during the sessions to capture key points and non-verbal cues.

To support participant comfort and openness during the interview process, participants were given a choice of interviewers. They could speak either with one of the group coordinators, with whom they had an existing rapport and might feel safer sharing their experiences, or with an independent third-party interviewer. This approach was designed to accommodate different preferences on how they would feel most comfortable discussing their experience. Most participants chose to be interviewed by a group coordinator.

Each interview began with an open-ended question designed to encourage reflection: ‘What was your experience with the group therapy?’ From there, participants were guided through a series of open-ended questions addressing the most valuable aspects of the group, areas for improvement and the broader impact of group dynamics on their experiences. The audio recordings were transcribed verbatim and imported into NVivo software for analysis.

#### Data analysis

Interview transcripts were examined using reflexive thematic analysis as outlined by Braun and Clarke (2006, 2019), guided by a critical realist stance. This perspective treats participants’ narratives as grounded in lived reality, while also recognizing that interpretation is inevitably shaped by the researchers’ assumptions and the broader sociocultural context in which the research is embedded (Wiltshire & Ronkainen, 2021).

The analysis was primarily inductive and focused on a semantic level of meaning, privileging participants’ explicit descriptions rather than searching for hidden or latent layers of interpretation (Braun & Clarke, 2019). This orientation was chosen to remain as close as possible to the language participants used when describing their experiences of the intervention.

The first author engaged in repeated readings of the transcripts to achieve familiarity with the data and generated initial codes to capture recurring patterns of meaning. Coding was treated as a reflexive process. Throughout this stage, analytic memos were kept to document evolving ideas, the researcher’s assumptions and decisions shaping the analysis (Chenail, 2015; Goundar, 2025; Mohajan & Mohajan, 2022). Attention was given to how the research team’s clinical training, neurotypical positionality and assumptions about therapeutic outcomes might influence interpretation. These issues were explicitly discussed in team meetings, which provided space for challenging assumptions and strengthening the analysis.

To enhance reflexivity, a second researcher coded a portion of the transcripts (25%). This step created a dialogic process in which alternative perspectives could be considered and coding decisions could be examined. In addition, a third team member reviewed the evolving thematic structure and provided feedback.

A former participant of the intervention and member of the research team joined the project as a co-researcher and co-author, contributing directly to the review and development of the themes. The construction of the themes was an ongoing collaboration and co-production between all authors, ensuring that lived experience meaningfully shaped the analytic process, representing participants’ voices authentically.

Theme development was a collaborative process. Initial codes were gradually clustered into broader patterns through ongoing discussion among the research team. Reflexive engagement was maintained throughout, with researchers critically considering how their professional and personal standpoints shaped the interpretations. The final themes provide a situated account of participants’ experiences of the group programme, illuminating both the benefits and challenges of engaging with the group therapy programme in the context of autism and social anxiety. Percentages were used descriptively to illustrate the spread of specific experiences within the sample, given the relatively large number of interviews. These figures were not used to imply statistical generalization, but to provide a broad representation of how commonly certain experiences were described.

### Community involvement

This study was co-produced in partnership with autistic individuals and neurodivergent researchers to ensure it aligned with community priorities and lived experience. A Community Consultation Committee, comprising autistic adults, including former participants of the CBT group programme, was actively engaged throughout the design and implementation processes, reviewing study aims, shaping the intervention format and advising on accessibility and relevance. In addition, a neurodivergent member of the research team, who was also a former participant, contributed as a co-researcher and co-author. Their role included co-designing the interview guide, interpreting qualitative data, collaborating on thematic analysis and reviewing manuscript drafts. This ongoing collaboration embedded lived experience into every stage of the project, from study design to dissemination.

## Results

**Figure 1.**
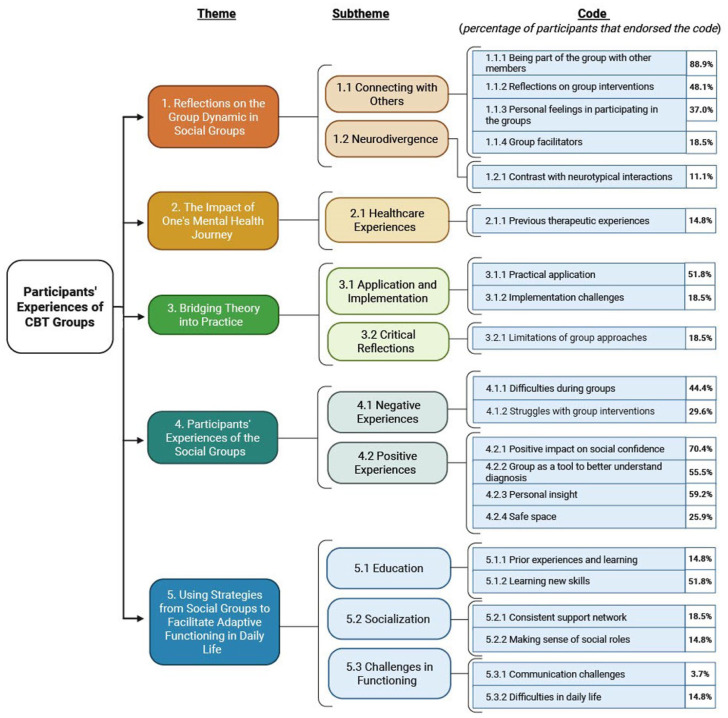
Results. Themes, subthemes and codes with the percentage of participants that endorsed the codes.

Thematic analysis revealed a range of primary themes, subthemes and specific codes (see Figure 1). The themes provide insight into different facets of the participants’ experiences with the modified CBT group intervention for social anxiety. Percentages were reported to provide a descriptive overview of how specific experiences were distributed across the sample. These figures are intended for illustrative purposes only and are not meant to imply statistical generalizability. We also provide examples of participants’ quotes during the interviews according to specific codes in Supplement Table 3.

## Reflections on the group dynamic in social groups

### Connecting with others

#### Being part of the group with the other group members

Over 88% of participants highlighted the value of engaging with other group members during the sessions. The shared experiences and mutual understanding fostered within the group were cited as crucial elements in their therapeutic process. Most participants (70%) reported that connecting with peers in the group significantly improved their engagement with therapy, emphasizing that connecting with other group members was the highlight of their experience, as it mitigated feelings of isolation and provided a sense of community. The group setting was also noted for its role in providing diverse perspectives that helped participants better understand themselves and others, increasing social confidence and self-understanding. Several participants (20%) also reported the importance of the supportive environment, as the group offered a space for shared experiences without judgment. Overall, participants frequently highlighted the connections formed with others as the most valuable aspect of their experience.

#### Reflections on group interventions

Approximately 48% of group members valued the customized nature of the interventions, especially how they addressed specific needs, such as communication preferences, flexibility in group structure and recognition of social challenges. The modifications in the group sessions, such as the use of visual representations, were seen as a significant strength, providing participants with practical strategies that resonated with their experiences. Participants noted that the adaptations made within the group sessions were not only helpful but also affirming, as they acknowledged the diversity of autistic experiences rather than expecting participants to conform to neurotypical norms.

Many participants found that the practical strategies offered, such as exposure and role-playing, were grounded in a real understanding of the social and sensory challenges they faced. The structured approach facilitated participants’ understanding and application of the strategies discussed, as one participant pointed out: ‘I think one thing was I think the sort of structure aspect like having regular opportunities to talk about issues to do with social anxiety, social confidence and neurodiversity, like having a regular space’ (Participant 1). Others noted that the inclusion of neurodiversity-affirming language and the flexibility to adapt tasks to individual needs made the experience feel more relevant.

#### Personal feelings in participating in the groups

Several participants (37%) described a sense of relief and comfort from being part of the group. Those feelings were accompanied by a renewed sense of control, belonging and being understood. The supportive nature of the group was a recurring theme, and participants noted that the group setting allowed freedom of expression that sometimes was not a feature of their past therapeutic experiences.

Despite these positive experiences, some participants (18%) found certain aspects of the programme emotionally challenging, such as the behavioural experiments. In addition, one participant mentioned that the logistics of attending sessions, such as travel to get to the in-person sessions, were draining, but the value of connecting with others made these efforts worthwhile.

#### Group facilitators

During the interviews, 18% of participants highlighted the critical role that facilitators played in the success of the intervention. One participant noted the emotional challenges they overcame during the programme and credited the facilitators with guiding the group through these experiences: ‘This experience was quite emotionally challenging [. . .], and the facilitators navigated us so kindly, graciously, and expertly through the process. [. . .] I hope they are aware of the impact they make and the comfort they provide [. . .]’ (Participant 2). The facilitators’ individualized support was viewed as beneficial, particularly in helping participants feel included and encouraged to participate.

### Neurodivergence

#### Contrast neurotypical interactions

Several participants (11%) noted the benefit of practising communication in a supportive, neurodivergent environment. The group setting allowed participants to engage more freely, as one participant reflected: ‘It was really interesting to be in a room with people who are just like me. . . It illuminates a lot about the way that I interact with neurotypicals’ (Participant 3). The connection with neurodivergent individuals, which was often a new experience for participants, helped them gain insights into their interactions and revealed the unique challenges of neurotypical social expectations. This validation was considered very important for addressing personal insecurities related to neurotypical standards and expectations.

## The impact of one’s mental health journey

### Healthcare experiences

#### Previous therapeutic experiences

Around 14% of the group members shared insights about their previous experiences with therapy, which influenced their engagement and expectations of the current modified CBT programme. For some, these past experiences had been disappointing or unfulfilling. For example, participants reported that the Engage Program provided a beneficial dynamic that contrasted with their past experiences with therapy, especially in terms of how feedback and concerns were handled. One participant appreciated the encouraging environment of the group, which allowed for open discussion and consideration of individual concerns, as this was a marked difference from their past experiences, where raising concerns sometimes felt unwelcome.

## Bridging theory into practice

### Application and implementation

#### Practical application

Participants frequently highlighted the value of applying theoretical concepts and practical tools from the programme into their daily lives. More than half of the participants described significant shifts in their social interactions, indicating the practical application and impact of the sessions in their lives. For example, one participant expressed how they had never previously considered ranking their anxiety levels but found this new technique beneficial in their day-to-day experiences.

The impact of the modified CBT group led to significant changes in participants’ lives. One participant described how the programme allowed them to reflect on their relationships through an autistic lens, leading to a ‘radical change’ in their routine. In addition, one participant found it valuable to revisit conversations and explore different perspectives, which made it easier for them to make sense of their social experiences.

#### Implementation challenges

In contrast to the practical applications of the programme, 18% of participants encountered several challenges when attempting to implement what they learned and practised during the intervention. Participants acknowledged that while they found the theoretical knowledge of the programme beneficial, they struggled to put it into practice. Some participants emphasized the challenge of applying the skills in highly anxiety-provoking situations that could not be easily replicated in a group setting, for example, difficult conversations with family members and coworkers. They expressed that the behaviour experiments might not fully address the complexities of real-life scenarios, especially those involving difficult conversations or critical feedback.

### Critical reflections

#### Limitations of group approaches

Participants expressed critical reflections about the limitations of the approaches provided in the programme (18%). While several aspects, such as practising conversation skills in a safe environment, were described as helpful, they were not experienced as fully meeting participants’ needs. For example, a few participants noted that while the intervention helped them to better understand their social interactions, this was often just one piece of a larger puzzle. They expressed that some approaches, such as behaviour experiments, though supportive in specific moments where they could challenge negative thoughts, did not always translate to more unpredictable or emotionally complex real-world situations. One participant reported that although they appreciated reflecting on certain feelings or thought patterns, the strategies felt ‘too narrow’ and lacked depth when it came to addressing long-standing emotional difficulties, such as deeply ingrained negative core beliefs or past experiences of social exclusion.

## Participants’ experiences of the social groups

### Negative experiences

#### Difficulties during groups

Almost half of the members of the group reported difficulties they encountered during the programme. Challenges related to the content and format of the group sessions, and the participants’ personal experiences. Some participants found the structure and delivery of the sessions difficult to follow due to the length of the sessions. Sensory issues also posed difficulties for some participants, for example, the room noises and bright lights. Specific activities, such as role-playing and the behavioural experiments, were described as particularly challenging. One participant shared, ‘Role-Playing stuff. . . I found it. . . a bit too difficult for me’ (Participant 4). Others expressed concerns about the approach to behavioural tasks, where participants would have to predict things that could go wrong during the activity, feeling that it fostered a negative mindset rather than building confidence. A small number of participants (7%) identified notable challenges in engaging with the therapeutic content due to their past experiences with therapy. One participant recounted a particularly difficult week and how it affected one of the behaviour experiments that we did in group:

That week, the timing was bad because I’d been involved in, like, a traumatic incident a few days ago. And I was even considering not coming to the group. And I worked up the courage to come, so I think that’s why it was so hard for me. So, I don’t think it has to do with the task design. It was more to do with me; I couldn’t benefit at that time. (Participant 1)

This shows how past negative experiences can exacerbate the challenges of therapy, making certain tasks or activities feel overwhelming.

#### Struggles with group interventions

Approximately 29% of participants described experiencing difficulties with aspects of the group intervention, particularly when certain activities did not align well with their personal histories or needs. For some, previous negative experiences with support services influenced how they engaged with the group. One participant shared that earlier interactions with structured programmes had left them feeling disillusioned and emotionally vulnerable. As a result, entering another group setting, even one designed to be supportive, was accompanied by feelings of apprehension and mistrust. This participant expressed a deep sense of uncertainty about whether the group would truly help, noting that many past interventions had not been effective for them. They described feeling a kind of ‘last chance’ pressure, worrying that if this group, too, did not meet their needs, they would be left without options. This sense of hopelessness and fear impacted their ability to fully engage in the programme.

### Positive experiences

#### Positive impact on social confidence

More than 70% of participants reported improvements in social confidence because of their experiences in the social groups. These improvements were attributed to various factors, including increased exposure to social interactions, enhanced self-awareness and the supportive environment provided by the group. The programme also facilitated a greater understanding of social interactions and personal capabilities. One participant reflected on the impact of the group on their confidence: ‘ I saw dramatic changes to my confidence and self-worth’ (Participant 5). This transformation enabled participants to engage more effectively in various social settings, including those that previously felt overwhelming.

#### Group as a tool to better understand diagnosis

More than half of the group members (55%) reported that the modified CBT group was important for deepening their understanding of their diagnosis and connecting with peers who had similar experiences. This reflects the group’s role in providing support and resources to navigate everyday challenges of a world that is shaped by neurotypical norms and expectations. In addition, the shared experiences of the group members provided valuable insights, as one participant stated, ‘Hearing everyone else’s experiences and their stories was really great. It helped me understand autism and the world we live in better’ (Participant 6).

#### Personal insight

A total of 59% of the participants reported gaining significant personal awareness through their involvement in the group therapy, which helped them better understand their behaviours, feelings and relationships. Interacting with peers was important to provide different perspectives, helping some participants reassess social situations when examining past experiences. In addition, participants noted that they gained new coping strategies from the group’s discussions. As one participant stated, ‘I feel the burden of not understanding oneself to be lifting for the first time, and this group played a significant part in this’ (Participant 2).

#### Safe space

The group was considered an important safe space that allowed for open experimentation and practice in a affirming environment for 25% of participants. One participant emphasized the value of having a safe space specifically designed for autistic individuals. The group also supported participants in facing and reframing their fears in a controlled setting, as one participant noted, ‘Supported opportunities to practice scary things were helpful . . .’ (Participant 1). This sense of being heard and validated was a key positive aspect of the group experience shared by many participants.

## Using strategies from social groups to facilitate adaptive functioning in daily life

### Education

#### Prior experiences and learning

Participants shared different reflections on their prior experiences and how these informed their learning and adaptation within the social groups. Almost 15% highlighted the sense of acceptance of past experiences, which have shaped their current understanding and coping mechanisms. Some participants had prior knowledge of the strategies presented, which was reinforced through the group. One participant mentioned, ‘On the content of being tactful, yeah, I already knew that before from the psychologist, and I find it helpful’ (Participant 7). This indicates that while the participant had previous knowledge, the group provided additional reinforcement of these strategies.

#### Learn new skills

More than half of the group members reflected on how the sessions introduced approaches that supported their existing strengths and offered practical strategies for navigating social situations. Several participants appreciated being introduced to alternative techniques that were tailored to autistic experiences. As one participant noted, ‘The different aspects of like . . . “this is how it’s regularly done, but for autistic people it’s commonly an issue because of these reasons . . .” I really got a lot out of that because . . . there are alternatives for me’ (Participant 8). This reflects the value of presenting strategies that align with autistic ways of being, rather than requiring individuals to conform to dominant social expectations. Others found the group’s structured format particularly helpful, noting that its clarity and predictability made it easier to engage with and apply social strategies in everyday contexts.

### Socialization

#### Consistent support network

Members of the group (18%) highlighted the value of having a reliable and structured support network as part of their social group experience. The regular opportunities to engage with peers and address challenges related to social interactions, interpersonal confidence and neurodiverse experiences provided a sense of stability and continuous support.

#### Making sense of social roles

A total of 14% of participants shared that the social groups supported their understanding of relationship dynamics and helped them reflect on how they relate to others in ways that felt authentic to them. One participant described how the group fostered conversations that empowered them to communicate more openly in their relationships: ‘Just sort of having that insight means I can open up dialogue with a friend or a partner or anything. And work through what works best with us’ (Participant 9).

### Challenges in functioning

#### Communication challenges

One participant reflected on the challenges of being misunderstood due to differences in their communication style:

Yeah, it tends to be the case that when people meet me, they find me unusual. I tend to leave quite strong first impressions that are usually negative, and then people just sort of go, ‘Oh, he’s not actually being rude, he’s just a bit loud and abrasive.’ (Participant 10)

This shows the impact of social assumptions and the need for greater understanding of diverse communication styles. The same participant also shared how they had developed their own communication strategies to navigate different social contexts, while acknowledging the difficulty of conforming to rigid, neurotypical conversational norms. Rather than indicating a deficit, this reflects the participant’s adaptability and the tension between authentic self-expression and external expectations.

#### Difficulties in daily life

A total of 14% of participants reported facing several significant difficulties in their daily lives, many of which stemmed from the challenges of navigating a world that often does not accommodate their unique needs and experiences. One participant described the effort required to manage day-to-day life due to the need to teach themselves various skills: ‘How many things don’t come naturally, which I’ve had to teach myself and learn how to navigate in day-to-day life’ (Participant 11)

The impact of societal norms on individuals with autism was also a concern. The same participant reflected on the traumatic effects of societal expectations: ‘Like a general awareness of how society can be quite traumatizing to people who are autistic purely because they’re autistic’ (Participant 11). This underscores the broader systemic challenges faced by autistic individuals. Participants also noted specific struggles with communication and emotional boundaries. In addition, the concept of ‘camouflaging’, where autistic individuals consciously adapt their behaviour to fit social expectations, was identified as a source of significant stress and fatigue.

## Discussion

This study provides valuable insights into the experiences of autistic adults participating in a modified CBT group therapy programme for social anxiety. The aim was to gather participants’ experiences on both the benefits and challenges of the modified group intervention programme, highlighting critical areas for future intervention design and implementation. The key findings of positive experiences were of the interactions with peers, being part of a neurodivergent community, the importance of tailored intervention to specific autistic needs and the opportunity to practice conversation skills and exposure in a safe environment. These reports suggest that the group environment and processes of the programme may further support inclusion and well-being for participants. As for the challenges, participants reported the importance of the impact of previous therapy experiences, the difficulties in addressing negative core beliefs and the challenges of the real-world application of the strategies presented in the intervention. While, on the one hand, the group programme fostered a safe place to interact with like-minded individuals, participants reported that translating and completing exposure tasks in typical real-world environments remains a challenge.

Participants had overall a positive experience with the group therapy setting, including interaction with peers, strengthened social confidence, gain of personal insights and the experience of being in a safe space and seeing the group as a tool to better understand their diagnosis. An important theme that emerged from the interviews was the transformative impact of connecting with other autistic individuals. Most of the participants described that being part of a neurodiverse community was valuable, provided a sense of belonging and reduced feelings of isolation. This aligns with prior research suggesting that autistic individuals often benefit from shared understanding and mutual support in neurodivergent groups (Crompton et al., 2020).

Another aspect of being part of a neurodivergent environment was the space to interact without the pressure of conforming to neurotypical social norms and expectations. Many participants shared that the prior social interactions they had with neurotypical peers had been draining, due to the need to mask or adapt their behaviours to fit these expectations. In contrast, the group offered an opportunity for authentic communication in an environment free of judgement. This also helped participants reflect on their social interactions. They shared the value of observing and learning from others in the group. These experiences align with prior research suggesting that autistic individuals experience less social anxiety in neurodivergent groups, where interactions are more accommodating and can be more honest (Black et al., 2022; Crompton et al., 2020; Heasman & Gillespie, 2018; Milton, 2012; Shea et al., 2022).

Furthermore, sharing experiences within the group helped participants recognize that their challenges are common and valid, reducing stigma and negative self-perceptions. Being part of a group with other autistic peers who share similar experiences allowed participants to foster a sense of connection and validation. As one participant put it, ‘Seeing how others manage things made me feel less alone. It helped me understand myself better’ (Participant 2). This suggests that neurodivergent peer support can play a crucial role in therapy, shifting self-perception from a deficit-based view to one that embraces neurodiversity. This aligns with research from Wilson (2024), where autistic individuals found peer recommendations and experiences more relatable and immediately applicable.

While the group aspects of the programme were considered beneficial by most participants, several challenges were reported, particularly related to sensory sensitivities and maintaining focus during the sessions. Some participants found it difficult to sustain attention for extended periods, while others struggled with environmental factors such as bright lighting and background noise, which caused discomfort, distraction and affected their engagement with therapy. These findings align with research indicating that sensory processing differences in autism can heighten arousal and intensify sensitivity to social stimuli (Baranek et al., 2005; Ben-Sasson et al., 2008; Leekam et al., 2006). Although facilitators attempted to mitigate this by adjusting blinds, offering flexible seating and providing access to quieter spaces where feasible, the setting posed limitations that could not be fully addressed. These sensory barriers may have influenced participants’ ability to engage and highlight an important area for improvement in the design of future interventions. In addition, navigating social environments can be particularly challenging for autistic individuals when sensory overload interferes with their ability to engage in meaningful interactions (Black et al., 2022). Some participants noted that prolonged social engagement itself during the sessions was draining, stating that they felt cognitive and emotional fatigue after the group sessions. During the sessions, it was discussed strategies the participants found personally restorative and exchanged ideas with peers about how to recover from the exhaustion associated with social interactions in the group setting.

The impact of the participant’s previous mental health journey on therapy was an important theme that emerged. Participants with negative prior therapy experiences said that they approached the group intervention with heightened scepticism and resistance. This individual context can influence their perception of the intervention and its effectiveness (Menezes et al., 2022; Wilson & Gullon-Scott, 2024).

In addition, some participants noted that while the strategies explored in the group were helpful, applying them in everyday life was often more complex and nuanced. Social situations outside the structured, supportive environment of the group involved a range of unpredictable dynamics, such as navigating workplace interactions and demands, managing emotionally charged conversations or interpreting nonverbal communication across different social contexts. Participants expressed that although the group felt like a safe and affirming space to practise these strategies, it did not fully reflect the reality of social interactions they faced outside the sessions. Several participants suggested incorporating more experiential components, such as more role-playing or scenario-based discussions, to build confidence in applying strategies meaningfully in their own lives. Rather than ‘teaching’ social skills, these activities were described as helpful for increasing comfort and self-understanding in social engagement. Future programmes might consider integrating potentially more flexible and adaptable tools, using media like virtual reality (Dechsling et al., 2021), or in vivo support with therapists in real-world environments. This may further assist with generalization and contextual adaptation, while maintaining a neurodivergent-affirming framework.

The importance of addressing communication challenges and social expectations specific to autism emerged as a critical theme in participants’ experiences. Many pointed out the value of interventions tailored to neurodiverse communication styles, specifically the theme of setting boundaries, and the influence of societal expectations. For instance, one participant noted the practical benefits of learning ‘I statements’, which enhanced their ability to navigate emotionally challenging conversations. This aligns with findings from Wilson and Gullon-Scott (2024), which emphasize that while autistic individuals benefit from therapy adaptations to improve accessibility, such modifications are often lacking in current clinical practice (Brice et al., 2021; Spain & Happé, 2019).

Moreover, Wilson and Gullon-Scott (2024) highlight that addressing specific communication needs in therapy can be a learning opportunity for both the client and therapist. The concept of the ‘double empathy problem’ (Milton, 2012) is particularly relevant in this context, as mismatched communication styles between autistic clients and neurotypical therapists may create misunderstandings or barriers to engagement. This demonstrates the necessity of equipping therapists with a deeper understanding of neurodivergent communication and ensuring that interventions foster mutual comprehension rather than placing the onus of adaptation solely on the autistic individual.

These findings align with the recent literature that states that CBT for social anxiety typically focuses on offering support for individuals to re-evaluate fears of negative evaluation. However, when these fears are well-grounded in actual experiences, such as bullying, exclusion and abusive relationships, this approach may be less effective and potentially invalidating (Wilson, 2024; Wilson & Gullon-Scott, 2024). Several participants in the group programme reported difficulties engaging with discussions or activities aimed at exploring and reframing negative social beliefs. For these individuals, the cognitive restructuring part of the programme could create an expectation to ‘think differently’ about their fears felt misaligned with their personal histories, where social rejection had been a consistent reality. This might have made it difficult for some participants to fully engage in certain parts of the group process that focused on challenging self-doubt, as these approaches could feel dismissive or emotionally unsafe.

These reflections show the urgent need for social anxiety interventions to acknowledge and directly address the impact of discrimination and interpersonal invalidation, which may shape autistic people’s social fears and sense of self. Rather than framing these fears as distorted or irrational, interventions might benefit from including strategies that validate these experiences and offer tools for managing their social and emotional consequences. Notably, participants found moments of support and perspective-sharing from fellow group members especially valuable in this regard. When encouragement or alternative viewpoints came from peers with similar experiences, the process felt more validating and meaningful. This peer-based support appeared to offer a sense of shared understanding that some participants felt had been lacking in prior therapeutic contexts, where clinicians may have minimized or misunderstood the legitimacy of their social fears.

Our findings offer specific insight into what neurodivergent-centred therapeutic spaces can look like in practice. Participants consistently described the group as a space where they could interact with peers without needing to mask or conform to neurotypical expectations. The experience of being among other autistic adults provided an affirming space and allowed for more authentic engagement in therapy. Key adaptations that contributed to this validating environment included a predictable session structure, low sensory-demanding settings, transparency from facilitators and encouragement to engage in ways that felt comfortable (e.g. taking breaks, using preferred communication styles). Importantly, the group allowed participants to approach exposure tasks with a sense of autonomy and support, rather than pressure. These elements were not framed as ‘training’ participants to behave neurotypically, but as scaffolding to help them explore social situations in ways aligned with their values and goals. Together, these findings provide a practical framework for designing interventions that are both effective and respectful of neurodivergent identities.

Taken together, these challenges underscore the importance of ensuring that interventions for autistic individuals are flexible, sensory-considerate and supplemented with long-term support structures (Benevides et al., 2020; Lei, Cooper, & Hollocks, 2024; Wilson, 2024; Wilson & Gullon-Scott, 2024).

### Limitations

Several limitations must be considered when interpreting these findings. First, we acknowledge that our sample is not generalizable to the broader autistic population. Participants had relatively high intellectual abilities and were all help-seeking individuals, interested in participating in a CBT group therapy for social anxiety. Reports do not reflect experiences from people with intellectual disability, from a broad age range and those from different cultural groups. In addition, we did not collect data on participants’ ethnicity, which limits our ability to comment on the cultural or racial diversity of the sample. Future research should aim to include and report on a broader range of demographic characteristics to enhance the inclusivity and generalizability of findings. Second, while the study provides rich qualitative data, its reliance on participant self-reports introduces the potential for recall bias. For instance, while some participants shared detailed insights about their prior therapeutic experiences, others provided limited information, which could affect the depth and consistency of the analysis. In addition, the 8-week intervention timeframe may have limited participants’ ability to reflect on longer-term outcomes of the intervention. We recommend that future research incorporate follow-up interviews with the participants. Third, this study did not systematically compare the modified CBT intervention to alternative therapeutic approaches, nor did it include the perspectives of facilitators or clinicians involved in the programme. These omissions limit our ability to evaluate the broader applicability and efficacy of the intervention. Fourth, participants were given the choice of an interviewer, and the majority opted to speak with a group coordinator rather than an independent third-party interviewer. We did this so that individuals could choose interviewers they felt most comfortable sharing insights with. While the choice of the group facilitator may have provided a sense of safety and comfort, it could also have introduced response bias, as participants might have been influenced by their existing relationship with the coordinator. They may have felt less inclined to be openly critical or to disclose certain experiences fully. Finally, it is important to note that the intervention was delivered in a group format, which, although well-received by participants, departs from standard recommendations for social anxiety treatment in the general population. National Institute for Health and Care Excellence (NICE, 2011, 2012, 2013) guidelines typically recommend individual CBT due to its greater cost-effectiveness and ease of implementation in routine clinical settings. Group-based interventions, while supported by evidence of therapeutic benefit, often face logistical and funding challenges that limit their feasibility in community practice. Nonetheless, our findings suggest that group-based CBT may be beneficial for autistic adults, including opportunities for peer connection, shared learning and engagement within affirming environments. These features align with NICE (2011, 2012) guidelines for autism, which advocate for flexible, person-centred and needs-led adaptations. Future research should further investigate the feasibility, sustainability and outcomes of group-based interventions within service models that support neurodivergent-affirming care. It is also important to acknowledge the accessibility limitations of group-based formats. Some autistic individuals who experience heightened discomfort in group settings may have chosen not to participate, leading to possible underrepresentation of their perspectives. However, we also created a safe and inclusive environment where individuals with no prior experience of speaking in groups were able to participate meaningfully. Nevertheless, self-selection bias cannot be fully ruled out. This underscores the need for future research to explore alternative formats, such as individual or online interventions, that may better accommodate diverse preferences and promote broader inclusion within neurodivergent-affirming care models.

## Conclusions and future directions

This study explored the experiences of autistic adults participating in a modified CBT group therapy for social anxiety, uncovering valuable insights into the benefits and challenges of such interventions. Participants highlighted the transformative impact of connecting with peers in a neurodiverse group, the importance of tailored strategies to address their needs and the limitations of short-term therapy in addressing deeply ingrained social and emotional challenges. Together, these findings emphasize the critical role of creating neurodivergent-centred therapeutic spaces that foster community, validation and mutual support and highlight the benefits of integrating identity-affirming and peer-based approaches into clinical practice.

Future adaptations of social anxiety interventions for autistic adults should consider the impact of discrimination, social invalidation and past negative life experiences. Programmes may benefit from integrating components that explicitly acknowledge the legitimacy of these experiences and provide strategies for navigating a world that often responds negatively to neurodivergent communication and behaviour. This could include modules on coping with stigma, building self-advocacy skills and fostering community connections that reinforce autistic identity and resilience. Future interventions should continue to adopt a more flexible and collaborative approach to accommodate the needs and lived experiences of participants. First, modifying the sensory environment, such as reducing room lighting, background noise and other sensory triggers, may help participants feel more comfortable and able to engage meaningfully during sessions. In addition, changes to the structure and pacing of sessions, including offering shorter sessions, more frequent breaks or extending the programme over a longer time frame, could support sustained engagement while minimizing cognitive and sensory overload. Beyond structural and environmental considerations, the content of interventions should also be adapted to acknowledge and address the social realities faced by autistic individuals, particularly the experience of systemic discrimination and repeated social rejection. Incorporating strategies for coping with invalidation, building resilience and navigating social environments that may not accommodate neurodivergent communication styles can help ensure interventions feel validating. Finally, embedding peer-led or co-facilitated components may enhance the sense of psychological safety and shared understanding within the group, offering a more meaningful therapeutic experience. These adaptations represent important directions for future programme development and research.

## Supplemental Material

sj-docx-1-aut-10.1177_13623613251377930 – Supplemental material for Autistic adults’ experiences of cognitive-behavioural group therapy for social anxiety: Relational experiences of participation Supplemental material, sj-docx-1-aut-10.1177_13623613251377930 for Autistic adults’ experiences of cognitive-behavioural group therapy for social anxiety: Relational experiences of participation by Bruna B Roisenberg, Kelsie A Boulton, Emma E Thomas, Nina Perry, Dorothy Yu and Adam J Guastella in Autism
