# Supplementary material for: Autistic adults’ experiences of cognitive-behavioural group therapy for social anxiety: Relational experiences of participation
Source: Autism. 2025 Oct 18;30(1):197–212. doi: 10.1177/13623613251377930 (PMC12717296; doi:10.1177/13623613251377930)
Supplement: sj-docx-1-aut-10.1177_13623613251377930 – Supplemental material for Autistic adults’ experiences of cognitive-behavioural group therapy for social anxiety: Relational experiences of participation [file sj-docx-1-aut-10.1177_13623613251377930.docx]

**Supplementary Materials**

**Table 3**

**Participants' quotes according to codes.**

| **Theme** | **Subtheme** | **Code Description** | **% of Participants** | **Example Quote(s)** |
| --- | --- | --- | --- | --- |
| **1. Reflections on the Group Dynamic in Social Groups** | 1.1 Connecting with Others | 1.1.1 Being part of the group with other members | 88.9% | "I really liked when we got to engage with the other group members a lot..." (Participant 11); "I learned so much from my peers in the discussions in the group." (Participant 2) |
|  |  | 1.1.2 Reflections on group interventions | 48.1% | "I really appreciated how the program addressed issues that are common for autistic individuals." (Participant 8); "Having regular opportunities to talk about issues..." (Participant 1) |
|  |  | 1.1.3 Personal feelings in participating in the groups | 37.0% | "This relief is recalled often as it continues to permeate each day..." (Participant 2); "This experience was quite emotionally challenging..." (Participant 2) |
|  |  | 1.1.4 Group facilitators | 18.5% | "I was sitting with one of the facilitators... she encouraged me..." (Participant 1) |
|  | 1.2 Neurodivergence | 1.2.1 Contrast with neurotypical interactions | 11.1% | "Having a safe environment with other autistics..." (Participant 3); "It was really interesting to be in a room with people who are just like me..." (Participant 3) |
| **2. The Impact of One's Mental Health Journey** | 2.1 Healthcare Experiences | 2.1.1 Previous therapeutic experiences | 14.8% | "I've never felt growth..." (Participant 12); "I haven't seen that before within my own past personal therapies." (Participant 6); "You were really taking on board our concerns..." (Participant 1) |
| **3. Bridging Theory into Practice** | 3.1 Application and Implementation | 3.1.1 Practical application | 51.8% | "I find that I use [anxiety ranking] mentally now, like just day to day." (Participant 3); "...especially helpful in workplace situations." (Participant 6) |
|  |  | 3.1.2 Implementation challenges | 18.5% | "Though I have not implemented any of it, even just the knowledge... is helpful." (Participant 13) |
|  | 3.2 Critical Reflections | 3.2.1 Limitations of strategies | 18.5% | "It's helpful, but... I definitely need other things..." (Participant 6) |
| **4. Participants' Experiences of the Social Groups** | 4.1 Negative Experiences | 4.1.1 Difficulties during groups | 44.4% | "I've had some troubles... with sensory issues..." (Participant 14); "Role Playing stuff... I found it... a bit too difficult for me." (Participant 4) |
|  |  | 4.1.2 Struggles with CBT | 29.6% | "I personally really struggled with [CBT] because I have quite an intense trauma history." (Participant 11); "I felt deflated like I couldn't do this." (Participant 1) |
|  | 4.2 Positive Experiences | 4.2.1 Positive impact on social confidence | 70.4% | "Being able to connect with the other people here was most helpful..." (Participant 14); "I saw dramatic changes to my confidence and self-worth." (Participant 5) |
|  |  | 4.2.2 Group as a tool to better understand diagnosis | 55.5% | "This group gave me the opportunity to understand myself better..." (Participant 2); "Hearing everyone else's experiences... helped me understand autism..." (Participant 6) |
|  |  | 4.2.3 Personal insight | 59.2% | "The burden of not understanding myself is lifting..." (Participant 2); "I was highly anxious... but I found I could handle them..." (Participant 3) |
|  |  | 4.2.4 Safe space | 25.9% | "Having a safe environment with other autistics..." (Participant 3); "Supported opportunities to practice scary things..." (Participant 1) |
| **5. Using Strategies from Social Groups to Facilitate Adaptive Functioning in Daily Life** | 5.1 Education | 5.1.1 Prior experiences and learning | 14.8% | "I already knew that before from the psychologist and I find it helpful." (Participant 7); "Particularly using that in a workplace situation..." (Participant 6) |
|  | 5.2 Socialization | 5.1.2 Learning new skills | 51.8% | "Learning tips on how to manage [information trading] has been... most useful." (Participant 6); "There are alternatives for me." (Participant 8) |
|  |  | 5.2.1 Consistent support network | 18.5% | "Having regular opportunities to talk about issues... like having a regular space." (Participant 1) |
|  | 5.3 Challenges in Functioning | 5.2.2 Making sense of social roles | 14.8% | "Open up dialogue with a friend or partner..." (Participant 9) |
|  |  | 5.3.1 Communication challenges | 3.7% | "There’s a danger in trying to internalize conversation as a series of rules." (Participant 10) |
|  |  | 5.3.2 Difficulties in daily life | 14.8% | "Things don’t come naturally... I've had to teach myself..." (Participant 11); "Society can be quite traumatizing..." (Participant 11); "I struggled to voice my own boundaries."( Participant 11) |

*Note: Participant number in brackets.*

| **Code** | **Definition** |
| --- | --- |
| **Being part of the group with other members** | The importance of peer connection and sharing experiences with fellow group members during the modified CBT sessions and the creation of a support network. |
| **Reflections on group interventions** | Participants' thoughts on the interventions, emphasizing the benefit of customized techniques for autistic individuals. |
| **Personal feelings in participating in the groups** | Emotions experienced during group participation, highlighting comfort, relief, and emotional challenges. |
| **Contrast with neurotypical interactions** | The distinction participants made between interactions within a neurodivergent group versus those with neurotypicals, often favoring the former for comfort and understanding. |
| **Previous therapeutic experiences** | Participants' reflections on past therapy experiences and how they compared to the current CBT group. |
| **Practical application** | The ability of participants to apply theoretical concepts from the social confidence program to their daily lives. |
| **Implementation challenges** | Difficulties encountered when attempting to implement strategies learned in the group into real-world situations. |
| **Limitations of strategies** | Critical reflections on the limitations of the techniques provided in the program, with suggestions for further support. |
| **Difficulties during groups** | Challenges related to the format, content, and environment of the group sessions, including sensory issues, task difficulties, and challenges faced due to trauma histories. |
| **Struggles with CBT** | Participants' difficulties with CBT techniques, especially when they didn’t align with their personal experiences or needs. |
| **Positive impact on social confidence** | Improvements in social confidence as a result of participating in the group sessions. |
| **Group as a tool to better understand diagnosis** | The role of the group in helping participants better understand their autism diagnosis. |
| **Personal insight** | New insights gained through group participation that helped participants better understand their behaviors and feelings. |
| **Safe space** | The group provided a safe environment where participants could practice social skills without fear of judgment. |
| **Prior experiences and learning** | How participants' previous knowledge and experiences influenced their learning in the group. |
| **Learning new skills** | Acquisition of new strategies and techniques through group participation, specifically tailored to autistic individuals. |
| **Consistent support network** | The value of a reliable support network created through regular group meetings and discussions that has an impact on socialization. |
| **Making sense of social roles** | How the group helped participants better understand their social roles and navigate relationships. |
| **Communication challenges** | Struggles with communication, including being misunderstood and the effort required to manage social situations. |
| **Difficulties in daily life** | Challenges faced in daily functioning, including managing societal expectations and masking autistic traits. |
| **Cognitive restructuring** | Participants' varied experiences with this CBT technique, including both its benefits and limitations. (Redirecting, changing negative thinking patterns) |
| **Exposure as part of therapy** | Participants' experiences with exposure tasks, some finding them beneficial, while others found them anxiety-inducing. |
| **Speech task** | A specific exposure task involving public speaking, with mixed reactions from participants. |
| **Tools of anxiety management** | Strategies provided by the group to help participants manage their anxiety in social situations (ex: spoon theory, anxiety surfing). |
| **Social skills as part of therapy** | The role of social skills training in the group, with participants finding the structured approach helpful. (ex: entering conversations, maintaining conversations...). |
| **Suggestions for future groups + Trauma-informed therapy** | Recommendations for future iterations of the program, including trauma-informed care and practical role-playing activities. |
| **Personal experiences vs. group needs** | Participants' reflections on how well the program met their individual needs versus the group’s needs. |
| **Group facilitators** | The role of facilitators in creating a supportive and inclusive environment for the participants. |

**Example of how to code data:**

**1. Practical application**: The participant is reflecting on how awareness of triggers and coping strategies have become easier in real-world situations. This relates to applying the theoretical concepts learned in the group to manage anxiety effectively.

*"I'm aware of how I feel. In certain situations before entering the situation. Being aware of what triggers and stuff like that, which is then made coping easier."*

**2. Exposure as part of therapy**: The participant mentions being "pushed to do things" and realizing the situation isn't as bad as expected, which aligns with the purpose of exposure tasks in therapy. Exposure tasks help individuals face anxiety-inducing situations and learn that their fears may not match reality.

*"Being pushed to do things and then realizing it's not as bad as I think in my head that it will be."*

**3. Cognitive restructuring**: The phrase "mind over matter" indicates the participant is recognizing how their thoughts (anticipation of a situation) can exaggerate fear, and by confronting these situations, they realize it's their mind holding them back. This reflects cognitive restructuring, where participants challenge and reframe negative thoughts.

*"It's like your minds get stupid that when you do it, I guess it's sort of rewarding. To have done it. Because you wouldn't normally do it, so it's like you realize it's your mind holding you back. It is not as severe as you think it would be."*

**Interview questions:**

1. What was your experience with the group therapy?

What were the most valuable aspects of the group for you?

1. Were there any aspects of the group that you found less helpful or felt could be improved?
2. How effective were the exposure tasks in helping you manage social anxiety?

Can you describe any specific exposure tasks that stood out as particularly helpful or challenging?

1. How helpful was cognitive restructuring (redirecting or changing negative thinking patterns) in managing your social anxiety?

Were there any specific challenges or successes you experienced with this approach?

1. How useful did you find the education about autism and anxiety as part of the therapy?
2. How helpful was learning about social skills (e.g., starting conversations, assertiveness) during the group?
3. How important was being part of a group with other members?

Did the group dynamic influence your experience in any way?
